# Bacteriophages pass through candle‐shaped porous ceramic filters: Application for the collection of viruses in soil water

**DOI:** 10.1002/mbo3.1314

**Published:** 2022-09-20

**Authors:** Perrine Florent, Henry‐Michel Cauchie, Malte Herold, Leslie Ogorzaly

**Affiliations:** ^1^ Environmental Research and Innovation Department (ERIN) Luxembourg Institute of Science and Technology (LIST) Belvaux Luxembourg; ^2^ Faculté des Sciences, de la Technologie et de la Communication (FSTC), Doctoral School in Science and Engineering (DSSE) University of Luxembourg Esch‐sur‐Alzette Luxembourg

**Keywords:** bacteriophage, metagenomics, microbial ecology, transport

## Abstract

Despite the ubiquity of viruses in soils, their diversity in soil water has not been explored, mainly due to the difficulty of collecting them. In hydrology, soil water is usually collected using porous candles. This study proposes using these porous candles as a new tool for sampling viruses in soil water to analyze their passage through the ceramic part of the candles. The recovery of the viruses was determined after filtration under laboratory conditions using three model bacteriophages (MS2, ΦX174, and Φ6) and *Escherichia coli*, at neutral and acidic pH. Then, a field experiment was carried out where soil water filtration and viral identification by metagenomic shotgun were performed. At neutral pH, all bacteriophages tested successfully passed through the porous candles during the filtration process, with reductions of 0.02 log, 0.16 log, and 0.55 log for MS2 ΦX174 and Φ6, respectively. At pH 4.4, the passage of MS2 was not affected while ΦX174 underwent a slight reduction in recovery, probably caused by adsorption onto the filter material. Regarding the application of the porous candles in the field, the results obtained allowed the successful recovery of viruses, exposing porous candles as a new method suitable for the collection of viruses from soil water in the context of the study of viral communities.

## Graphical abstract

This study proposes to use porous candles as a new tool for collecting soil water viruses. In the first step, the passage of model viruses through the ceramic part of the candles was investigated. Regarding the field application, the experiments carried out showed a successful recovery of viruses, demonstrating the relevance and efficiency of such a tool for the collection of viruses from soil water in the context of metagenomic studies of viral communities.

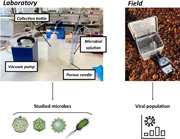

## 1 INTRODUCTION

Soil is a complex ecosystem composed of organic matter, minerals, air, and water. Although soil water accounts for about 20%–30% of the total soil composition, to date, it remains the least studied fraction (Kalev & Toor, 2018). Within soil environments, water freely circulates through soil pores, consisting of macro (>0.08 mm) and micropores (<0.08 mm), allowing the transport of water either between soil aggregates in the macropores or within aggregates in the micropores (Easton & Bock, 2016; Gardner, 1962). As water moves through the soil, it has the potential to carry most particles smaller than the soil pores with it, including viruses. The soil is very diverse and is the most abundant compartment of the biosphere for viruses (Kimura et al., 2008), whose abundance can reach up to 10^10^ particles per gram of soil (Ashelford et al., 2003; Kimura et al., 2008; Williamson et al., 2013). Viruses form an essential part of the microbial activities in soil and can be found in every type of soil, such as agricultural soils, forest soils, wetlands, or even cold deserts (Florent et al., 2022; Williamson et al., 2017). Bacteriophages are reported as being the most abundant viruses in soils, and thus, knowledge of soil viral diversity was gained by investigating bacteriophage diversity (Ashelford et al., 2003; Swanson et al., 2009; Williamson et al., 2017). Viruses are more stable in cool, and wet environments, resulting in higher viral abundance reported in wetlands than in forests, but lower in agricultural soils than in forests (Hurst et al., 1980; Schijven & Hassanizadeh, 2000; Williamson et al., 2017). Many abiotic factors may be involved in explaining the distribution, abundance, and diversity of viruses in soils, such as soil pH, cation availability, or organic matter (Cao et al., 2016; C. Wang et al., 2019). The role of viruses is now recognized, particularly in terms of the control of bacterial population growth or in the interaction within biogeochemical cycles (Braga et al., 2020; Trubl et al., 2018). Due to the electrical charges present on the surface of viruses, most of them can interact with soil particles, through the adsorption process. This process is defined as the attachment of viral particles to the surface of any type of molecule or material (Schijven & Hassanizadeh, 2000; Zemb et al., 2013). Adsorption by electrostatic forces is affected by the environmental pH and the surface charges of the molecules on which the viruses can adsorb (Flynn & Sinreich, 2010; Germann et al., 2002). The adsorption of viruses was reported to occur on mineral surfaces, such as saprolite, colloidal particles or even clay (Flynn et al., 2004; McKay et al., 2000). This has led investigations to focus preferentially on the soil matrix, rather than soil water (Braga et al., 2020; McLeod et al., 2004).

Soil water sampling is usually carried out by ceramic filtration using porous candles with the general aim of determining the chemical composition of the soil water (i.e., pesticide, trace metal, nitrogen contents) (Paul et al., 2003; Rawles & Brakensiek, 1982; Riekerk & Morris, 1983; Rosenqvist, 1959; Yang et al., 2013). This method is based on a suction mechanism, allowing water to flow through the pores of the candles and excluding any particles larger than the pore size (typically 2 µm). The pore size is an important parameter in water treatment applications. These ceramic filters proved effective in removing microbial contaminants, such as some bacteria, protozoa, and microbial cysts, but could not entirely remove viruses, due to them being much smaller than the pore size (Lelago Bulta et al., 2019; Nigay et al., 2020; Zereffa & Bekalo, 2017). Ceramic suction samplers were used in sewage‐contaminated soils (Wang et al., 1980) and the study of pathogenic virus transport in wastewaters (Lance & Gerba, 1984). In these studies, the ceramic samplers proved their efficiency in recovering viruses from tap and sewage waters moving through soils, under either saturated or unsaturated flows. However, soil water collection was carried out on soil columns constructed for laboratory experiments and no studies have sampled soil water in the field to recover natural populations of bacteriophages.

In this study, we propose using porous ceramic candles as a novel sampling method to collect viruses in soil water. However, the passage of viruses through the ceramic part of the candle is still not fully understood. Bacteriophages were reported to adsorb onto the positively charged materials added to the ceramic part of the candle (Brown & Sobsey, 2009; Michen et al., 2013). Therefore, this study aimed to determine whether viruses can pass through porous ceramic candles without this causing their loss. We assumed that the viruses were small enough to pass through the porous candle but that adsorption onto the ceramic part could interfere with their filtration. To evaluate the filtration process of viruses through porous candles, two complementary methodological approaches were set up. First, three bacteriophage models and one bacterium were selected based on their size, isoelectric point (pI), hydrophobicity, and shape to perform a series of in vitro experiments under controlled conditions. Subsequently, to verify the potential of porous candles to collect viruses directly from soil water, an *in situ* experiment was conducted with the collection of soil water samples from a forest in Luxembourg, called Weierbach. The samples were then subjected to shotgun metagenomic analysis to detect the presence of viral DNA and thus confirm the effectiveness of the candles in collecting viruses.

## 2 EXPERIMENTAL PROCEDURES

### 2.1 Laboratory experiments

#### 2.1.1 Bacteriophage strains, stock production, and quantification

To conduct the first set of experiments on porous candles, three bacteriophages were selected (MS2, ΦX174, and Φ6) depending on their size, isoelectric point (pI), hydrophobicity, and shape. *Emesvirus zinderi* (former MS2) is a small (28 nm), icosahedral, and highly hydrophobic ssRNA‐bacteriophage with a pI of 3.5 (Dedeo et al., 2011; McKay et al., 1993). *Sinsheimervirus ΦX174* (ssDNA bacteriophage) has almost the same morphology as MS2, with an icosahedron of 27 nm (Elhadidy et al., 2013). With a pI ranging from 6.6 to 6.8, ФX174 is one of the least electrostatic and hydrophobic bacteriophages known (Mi, 2020). Finally, *Cytovirus* Φ6 is a hydrophobic lipidic‐enveloped ssRNA‐bacteriophage, with a spherical shape of approximately 85 nm with a pI = 6.94 (Bamford et al., 1987; Fedorenko et al., 2020; Kozlowski, 2016).

The three bacteriophages were obtained from culture collections: *E. zinderi* (former *Escherichia coli* bacteriophage MS2, ATCC 15597‐B1, LGC), *Sinsheimervirus ΦX174* (ATCC 13706‐B1, LGC), and *Cytovirus Φ6* (DSM 21518, Deutsche Sammlung von Mikroorganismen und Zellkulturen). Also, MS2 was replicated using *E. coli* Hfr K12 strain (CIP 104130, Collection Institut Pasteur) as the bacterial host according to the ISO method 10705‐1:2001. Stocks of ΦX174 were produced with *E. coli* WG5 strain (CIP 107680, Collection Institut Pasteur), as described in the ISO method 10705‐2:2001. The replication of Φ6 was conducted using the bacterium *Pseudomonas* sp. (DSM 21482, Deutsche Sammlung von Mikroorganismen und Zellkulturen). The bacterial host was grown in 50 mL of TSB (Tryptone Soy Broth) for 18 h at 26°C, under agitation (110 rpm). A volume of 500 µL from the overnight culture was mixed with a fresh 50 mL of TSB for 5 to 6 h at 26°C, under agitation (110 rpm). Then, 1 mL of the stock suspension of bacteriophage Φ6 (>10^4^ PFU mL^−1^) was added to the bacterial vial and incubated at 26°C under agitation (110 rpm) until the lysis process was observed (overnight) (Pinheiro et al., 2020).

Following viral replication, bacteriophage suspensions were centrifuged at ×3000*g* for 20 min to remove the cell debris. The supernatant was then decontaminated by filtration using 0.22 µm membrane filters (MillexGP Millipore Express PES membrane, Millipore). Finally, the different bacteriophage stocks were stored at 4°C.

The bacteriophage stocks were enumerated using the double agar layer procedure according to the standard procedures ISO 10705‐1:2001 and ISO 10705‐2:2001 for MS2 and ΦX174, respectively. The plaque assay for Φ6 was conducted with the same protocol as for MS2 and ΦX174, using the *Pseudomonas* bacterial sp. (DSM 21482). Briefly, the host bacterium was grown on TSB for 18 h at 26°C, under agitation (110 rpm). Then, 500 µL of the previous culture was mixed with 50 mL of TSB, for 4 to 5 h (DO ≈ 0.1) and then incubated at 26°C, under agitation (110 rpm). So, 2.5 mL of ssTSA (semi‐solid Tryptone Soy Agar with 0.75% agar), 1 mL of *Pseudomonas*, and 1 mL of diluted Φ6 solution were mixed and poured onto the first layer of TSA (Tryptone Soy Agar with 1.5% agar). The Petri dishes were then placed into incubation at 26°C for 24 h. The concentrations of the bacteriophage stocks were 5.00 × 10^9^, 5.43 × 10^9^, and 3.90 × 10^6^ PFU mL^−1^ for MS2, ΦX174, and Φ6, respectively.

#### 2.1.2 *Escherichia coli* K12 strain, stock production, and quantification

In addition to the previous bacteriophages, *E. coli* K12 bacterial strain was selected to verify the effective exclusion of bacteria due to pore size. It is a Gram‐negative bacterium that belongs to the *Escherichia* genus. It has a rod‐shaped morphology and is about 2.0 μm long with a 0.25 to 1.0 μm diameter (Neidhardt & Kushner, 2017; Rogers & Kadner, 2020). The *E. coli* K12 strain was produced as described in the ISO method 10705‐1:2001, used to produce the bacterial host of the MS2 bacteriophages. The enumeration of *E. coli* K12 strain followed the culture‐based method described in the standard method ISO 10705‐1:2001. Then, the bacterial solutions were quantified and expressed in CFU mL^−1^ (colony forming units per mL). The concentration of the stock solution was estimated at 1.00 × 10^8^ CFU mL^−1^.

#### 2.1.3 Porous ceramic filtration: Experimental design

**Figure 1:**
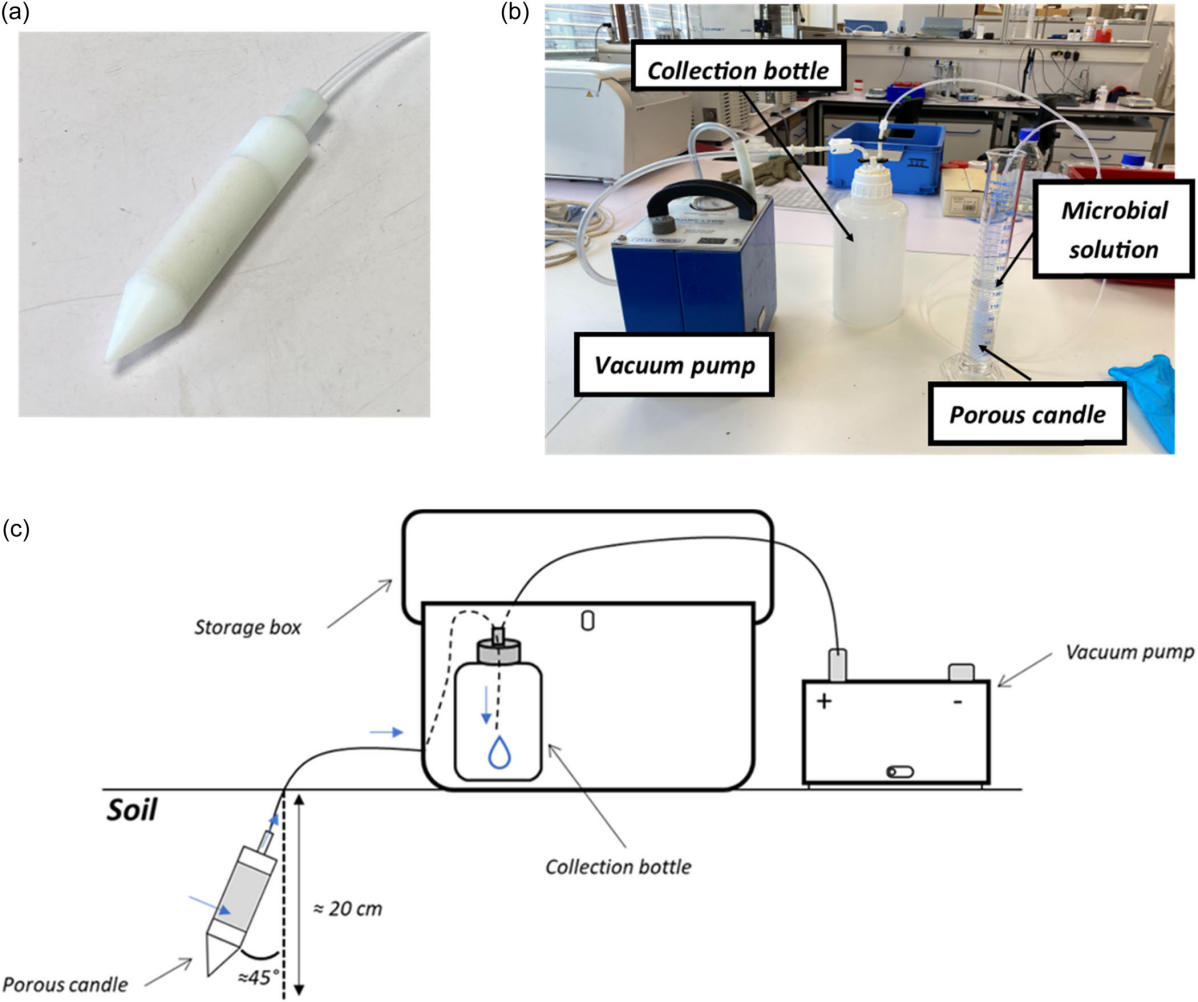
(a) Picture of a porous ceramic candle, (b) scheme of the experimental filtration design, and (c) scheme of the porous candle installed in soils, where the blue arrows show the transport of soil water from soil suction to sampling in the collection bottle.

The water was filtered using candle‐shaped filters (2440110, SDEC, Reignac sur Indre), with a diameter of 21 mm and a length of 95 mm (Figure 1a). The candles are made of polytetrafluoroethylene (or PTFE) covered with a quartz ceramic (silicon dioxide, SiO_2_), with a 2‐micron pore size. This porous ceramic candle has a porous area of 33 cm^2^ and hydraulic conductivity of 3.31 × 10^−7^ cm s^−1^. This type of candle was chosen as it does not retain any elements or chemical compounds from samples. Moreover, the chemical elements of the candles (e.g., aluminum, sodium, iron, zinc…) were quantified as being below the detection limits, so that they do not hinder the quality of the water samples (SDEC, 2002).

The porous candle was placed in a 100‐mL microbial solution (i.e., solution of MS2, Φ6, ΦX174, or *E. coli*) in a glass test tube and connected to a collection bottle via a plastic extension on a polyacetal (polyoxymethylene or POM) screw cap. The collection bottle was also connected to a P2000 electric vacuum pump with an automatic drive (1800000, SDEC, Reignac sur Indre). The pump was set to −0.8 bars to depressurize the collection bottle, thus generating the suction of the microbial solution through the porous candle by capillarity (Figure 1b).

Two experimental conditions were investigated to assess the pH effect on the filtration process through the porous candle: pH = 7.0 and pH = 4.4. For each experiment, 500 mL of microbial spike solutions (i.e., MS2, ΦX174, Φ6, and *E. coli* K12) were prepared with peptone saline water (pH 7.0 and pH 4.4) to reach a feed concentration of 10^6^ PFU mL^−1^ for MS2 and ΦX174, 10^3^ PFU mL^−1^ for Φ6 and 10^7^ CFU mL^−1^ for *E. coli* K12. Each experiment was performed in quadruplicate using four replicate porous ceramic candles. For the experiment at pH 4.4, only the bacteriophages MS2 and ΦX174 were tested. A pH of 4.4 was chosen because it is the lowest pH measured at the Weierbach site. At this pH, the net surface charges of ΦX174 became positive (pH below the pI of the bacteriophage), whereas MS2 remained negatively charged. The bacteriophage solutions were acidified using hydrochloric acid (HCl, pH = 3.1). The microbial feed solutions, as well as the permeate solutions, were enumerated by the procedures previously described (see Sections 2.2 and 2.3), and the Log Reduction Value (LRV) was estimated for each experimental condition as follows:

$$\text{LRV}\;=\log\left(\frac{C_{p}}{C_{f}}\right)$$

where *C*_f_ and *C*_p_ are the concentration of microorganisms in the feed solution and permeate, respectively.

### 2.2 Field experiments

#### 2.2.1 Study site and soil water collection

The field campaign was carried out on the Weierbach plateau (latitude: 49°83'46''55'''N, longitude: 5°79'64''27'''E) and took place in November 2020. The Weierbach is a subbasin located northwest of the Attert River basin, in the Grand Duchy of Luxembourg. The subbasin has an area of 0.45 km² and belongs to the Ardennes massif in the Oesling region (Martínez‐Carreras et al., 2012). The five porous candles used in the previously mentioned lab experiments were randomly installed at 20‐cm soil depth in a delimited area of 25 m^2^ on the Weierbach plateau. The candles were then connected to both a collection bottle and to a P2000 electric vacuum pump, as with the lab experiments (Figure 1c). The pump was set to −0.8 bars to trigger the collection of soil water. Since the aspiration process took approximately 24 h, soil water samples were recovered from the collection bottle on the next sampling day. In addition, the volume of water collected using the porous candles in the field is often uncertain, thus, the soil water recovered in the five collection bottles was pooled to increase the final volume. Ultimately, a total of six pooled replicates were collected with final volumes ranging from 43 to 165 mL. The pH of each soil water sample was measured, and the samples were then stored at 4°C before further analyses.

#### 2.2.2 The concentration of soil water samples

This step aimed to increase the concentration of viral particles from the soil water samples collected directly in the field using a laboratory enrichment protocol. For this purpose, a primary concentration step using a Centricon® plus‐70 centrifugal ultrafilter with a cut‐off point of 10 kD (UFC710008, Millipore) was performed at ×3200*g* for 20 min, at 4°C on volume samples ranging from 43 to 165 mL, according to the manufacturer's recommendations. This step resulted in a final volume of approximately 4 mL for all samples. A secondary concentration step was carried out using an Amicon Ultra‐4 10 kD (UFC801024, Millipore) at ×4000*g* for 20 min, at 4°C, according to the recommendations of the manufacturer, to reach this final volume (200 µL) for all samples.

#### 2.2.3 Total DNA extraction and shotgun metagenomics sequencing

Total DNA extractions were performed on the 200 µL of soil water concentrates, using the DNeasy® PowerSoil® kit (Qiagen). The protocol followed the manufacturer's recommendations: cell lysis, followed by DNA fixation and elution. However, the recommended volume of 100 μL for the elution step was reduced to 60 μL to concentrate the DNA. Final DNA concentrations were quantified using the Invitrogen™ Qubit™ 2.0 fluorometer (Thermo Fischer) and ranged from 0.37 to 1.0 ng µL^−1^. The extracted DNA was then stored at −20°C until further analyses. The viral communities were determined using shotgun metagenomics sequencing. From the total extracted DNA of soil water samples, library preparations were completed using the Nextera DNA Flex kit (Illumina). Paired‐end sequence reads were generated using the Illumina NovaSeq. 600 (2 × 150 bp).

#### 2.2.4 Reference‐based bioinformatics

The raw sequencing reads were quality‐controlled (sliding window of 4 and minimum quality score of 20, minimum read length of 100 bps) and Illumina adapters (NexteraPE‐PE. fa) were trimmed with Trimmomatic v0.39 (Bolger et al., 2014). Assemblies were performed separately for each sample using SPAdes v3.15.4 (Nurk et al., 2017) with the “meta” option and default parameters. Taxonomy classification of contigs was carried out against the complete nonredundant (nr) microbial protein sequences database (released version of April 2022, downloaded in May 2022) from NCBI using MMseqs. 2 v13.45111 (Steinegger & Söding, 2017) and the easy‐taxonomy workflow that determines ancestors by searching translated ORFs (translation table 11) (Mirdita et al., 2021). Quality‐filtered reads from each metagenome were mapped back to the assembled contigs using minimap2 v2.24 with the “sr” preset (Li, 2018), then sorted and indexed with SAMtools v1.15.1 (Danecek et al., 2021). Finally, metagenomic profiles were exported as comma‐separated value (csv) files. Relative abundances were calculated based on the number of reads mapping to each contig. Mapped reads were divided by contig length and the total number of mapped reads per sample (Palermo et al., 2019; Zhao et al., 2021).

A mathematical transformation of the relative abundances of the microbial species was performed to normalize the replicates since the volumes recovered were different for each candle. The transformation was performed as followed:

$$\mathrm{At}=\frac{A\times r}{V_{i}}$$

where *At* is the transformed relative abundance in 1 mL of sample, *A* is the relative abundance, *r* is the ratio of the volume (µL) extracted after DNA extraction to the volume (µL) analyzed during metagenomic sequencing, and *V*_i_ is the initial volume (ml) collected for each soil water sample.

### 2.3 Statistical analyses

Statistical analyses were performed with RStudio, version 1.4.1106, and R version 4.0.4 (Rstudio Team, 2020). The figure was generated from the percentage of the transformed relative abundance using the package “ggplot2,” “ggpubr,” and “cowplot.” Nonparametric tests (Kruskal–Wallis One Way ANOVA on rank) were used followed by Dunn's test (multiple pairwise comparisons) with Holm's correction to compare the microbial concentrations before and after filtration through the porous candles. These statistical tests were performed separately for the experiments at pH = 7 and pH = 4.4 (*N* = 32 and *N* = 16, respectively).

## 3 RESULTS

### 3.1 Laboratory experiments

The results regarding the filtration of all microorganisms at pH 7.0 are reported in Table 1. For bacteriophages MS2, ΦX174, and Φ6, 100 mL solutions with the respective feed concentrations of 3.65 × 10^6^, 1.55 × 10^6^, and 2.44 × 10^3^ PFU mL^−1^ were brought into contact with the porous candles. After filtration through the candles, the permeate concentrations were 3.48 × 10^6^, 1.07 × 10^6^, and 6.88 × 10^2^ PFU mL^−1^ for MS2, ΦX174, and Φ6, respectively. For all bacteriophage solutions, no statistical difference was observed between the permeates and the feed solutions (Kruskal–Wallis test: *p* = 0.7622 for MS2, *p* = 0.8175 for ΦX174, and *p* = 1 for Φ6, at *α* = 0.05). The filtration process induced an LRV of 0.02 log, 0.16 log, and 0.55 log for MS2, ΦX174, and Φ6, respectively.

**Table 1:** Estimated concentrations of microorganisms in feed solutions and permeates in porous candle experiments conducted at pH 7.0 and pH 4.4, and their associated LRVs

	pH 7.0			pH 4.4		
Microorganisms	Feed solution	Permeate (*n* = 4)	LRV	Feed solution	Permeate (*n* = 4)	LRV
	Concentration + CI (PFU ml^−1^) or (CFU ml^−1^)	Median concentration + IQR (PFU ml^−1^) or (CFU ml^−1^)	Median + IQR	Concentration + CI (PFU ml^−1^) or (CFU ml^−1^)	Median concentration + IQR (PFU ml^−1^) or (CFU ml^−1^)	Median + IQR
MS2	3.65 × 10^6^ ± 3.50 × 10^5^	3.48 × 10^6^ ± 8.38 × 10^5^	−0.02 ± 0.06	1.63 × 10^6^ ± 4.50 × 10^4^	1.25 × 10^6^ ± 2.24 × 10^4^	−0.11 ± 0.01
ΦX174	1.55 × 10^6^ ± 2.50 × 10^5^	1.07 × 10^6^ ± 2.39 × 10^5^	−0.16 ± 0.09	3.60 × 10^5^ ± 1.00 × 10^4^	1.01 × 10^4^ ± 9.05 × 10^3^	−1.57 ± 0.42
Φ6	2.44 × 10^3^ ± 1.50 × 10^1^	6.88 × 10^2^ ± 1.89 × 10^2^	−0.55 ± 0.12	ND	ND	ND
*Escherichia coli* K12	7.07 × 10^7^ ± 6.00 × 10^5^	1.50 × 10^4^ ± 1.13 × 10^5^ a	−3.80 ± 2.72	ND	ND	ND

Abbreviations: CFU ml^−1^, colony forming unit per milliliter; CI, confidence interval; IQR, interquartile range; LRVs, Log Reduction Values; ND, not determined; PFU ml^−1^, plaque forming unit per milliliter.

^a^ Significatively different from the feed solution for 0.01 < *p* < 0.05.

For *E. coli* K12 bacterial strain, the median permeate concentration (1.50 × 10^4^ CFU mL^−1^) was significantly different from that of the feed solution (7.07 × 10^7^ CFU mL^−1^) after filtration (Kruskal–Wallis test: *p* = 0.0113, *α* = 0.05). Furthermore, of the four candles used, the LRV of *E. coli* K12 ranged from 2 log to 6 log (−3.8 log median), showing variability in the retention of *E. coli* K12 in the filter.

For the second set of experiments, bacteriophages MS2 and ΦX174 solutions were prepared at pH = 4.4 and underwent passage through four different porous candles as previously described. The initial concentrations of the MS2 and the ΦX174 solutions were 1.63 × 10^6^ and 3.60 × 10^5^ PFU mL^−1^, respectively. After passing through the porous candles, the concentrations were 1.25 × 10^6^ and 1.01 × 10^4^ PFU mL^−1^ for MS2 and ΦX174, respectively (Table 1). No statistical difference between the concentrations before and after filtration was observed (Kruskal–Wallis test: *p* = 0.3412 for MS2 and *p* = 0.1137 for ΦX174, *α* = 0.05).

At pH 7.0, MS2 and ΦX174 bacteriophages showed a LRV of 0.02 and 0.16, respectively. At pH 4.4, the LRV was 0.11 for MS2 bacteriophage, while the LRV of ΦX174 was 1.57. The results for MS2 and ΦX174 did not reveal a significant reduction after the acidification of the bacteriophage solutions (Kruskal–Wallis test: *p* = 0.2045 and 0.1363, *α* = 0.05, respectively).

### 3.2 Total endogenous DNA viral populations recovered from collected soil water

**Figure 2:**
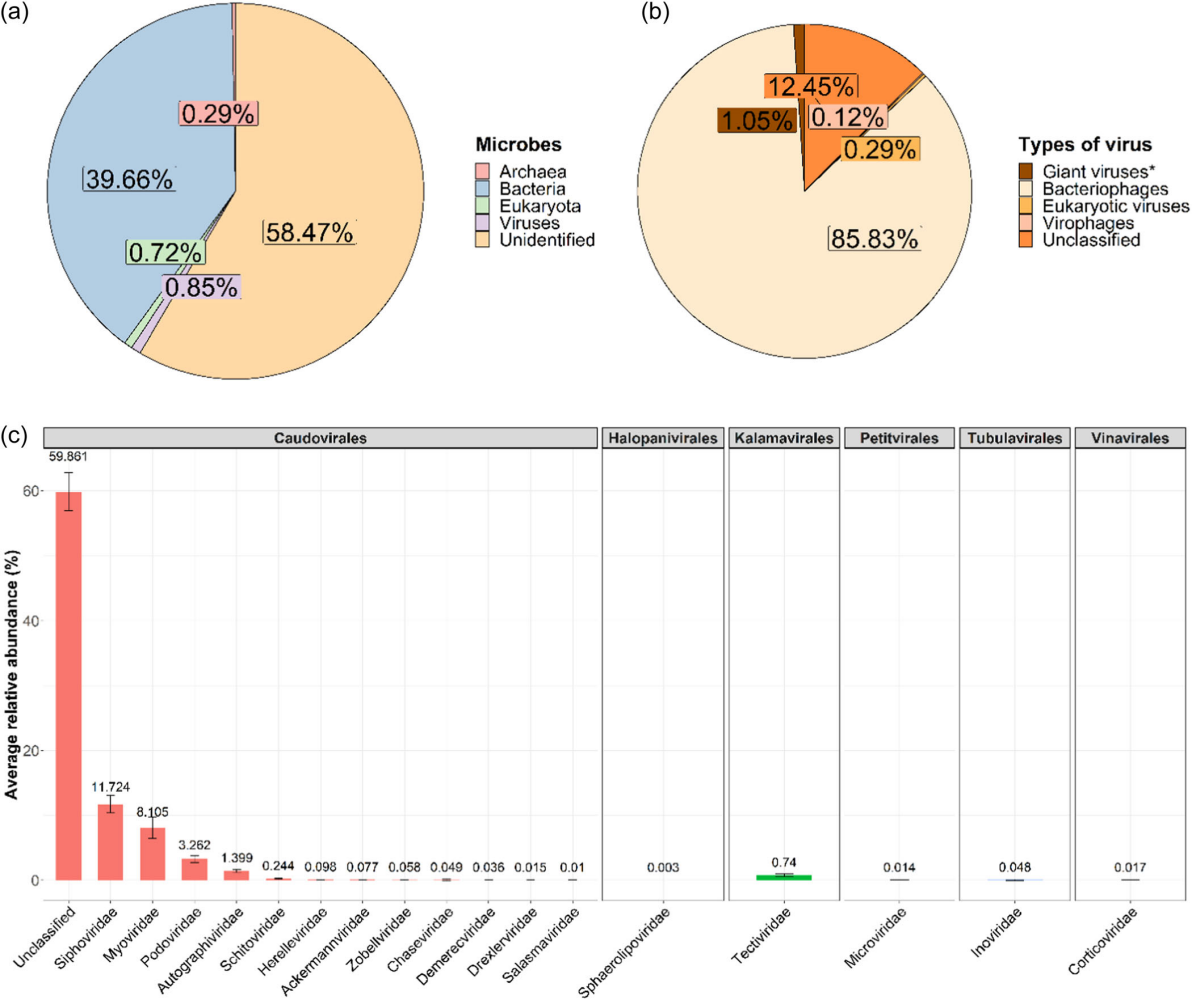
Pie charts of the relative abundances of (a) the total microbial populations and (b) the total viral populations recovered from soil water samples (*n* = 6) collected by the porous candles. (c) Bar chart representing the relative abundance of each DNA‐bacteriophage family comprising the soil water samples (*n* = 6).

The statistics of the metagenomic shotgun sequencing are reported in Table 2. Shotgun sequencing produced approximately 50 million paired reads and of these, 300,000 to 1.5 million contigs were created after assembly. Using the nr (non redundant) database and after *e*‐value filtering, approximately 30%–45% of the contigs were successfully identified. Of the identified fraction, on average, 95.5% of the sequences were identified as bacterial genomes, and the second most identified microbes were viruses with an average of 2.04% viral genomes (Figure 2a). These viral genomes were mostly represented by DNA‐bacteriophages (85.83% of the total viral sequences), while 1.05% represented giant viruses (all of them were nucleocytoplasmic large DNA virus, NCLDV), 0.29% eukaryotic viruses, 0.12% virophages and the remainder of the sequences (12.45%) were unclassified viruses (Figure 2b). While exploring viral composition in detail, a total of 38 viral families were identified, of which 18 were bacteriophage families (i.e., 95.05% of the viral sequences) and 13 belonged to the *Caudovirales* order. The high proportion of this order was mainly the result of a large abundance of unclassified *Caudovirales* (59.86%), *Siphoviridae* (11.72%), *Myoviridae* (8.10%), and *Podoviridae* (3.26%). In addition, five other families were identified: *Sphaerolipoviridae* (0.003%, *Halopanivirales* order), *Tectiviridae* (0.74%, *Kalamavirales* order), *Microviridae* (0.014%, *Petitvirales* order), *Inoviridae* (0.048%, *Tubulavirales* order), and *Corticoviridae* (0.017%, *Vinavirales* order) (Figure 2c).

**Table 2:** Statistics of the metagenomic sequencing: raw data, assembly, and mapping on the soil water samples

Soil water replicates	Number of read‐pairs	Yield in Mbp	Average quality (*Q*‐score)	Number of total contigs	GC‐content	N50	Identified total sequences^a^
SW1	52,568,931	14,966	35.70	748,555	57.47%	547	37.04%
SW2	50,275,130	14,523	35.62	437,122	56.15%	358	41.22%
SW3	53,588,349	15,350	35.56	675,363	56.33%	535	44.66%
SW4	48,195,690	13,687	35.81	1,675,417	57.18%	496	38.58%
SW5	48,213,814	13,625	35.59	1,813,938	55.99%	476	37.42%
SW6	51,119,776	14,665	35.47	1,837,051	56.95%	463	33.99%

*Note*: Total references to the total microbial metagenome (i.e., eukaryote, archaea, bacteria, and viruses).

Abbreviations: GC, guanine cytosine; Mbp, millions of base pairs; N50, the sequence length of the shortest contig at 50% of the total genome length.

^a^ Calculations based on the number of total contigs.

## 4 DISCUSSION

In the investigation of the passage of viruses through porous candles to be collected from soil water, the model bacteriophages selected in this study succeeded in demonstrating this process. In the study of Van der Laan et al., bacteriophages showed lower retention than *E. coli* bacteria at a neutral pH (≈−0.6 log for MS2 vs. ≈−1 log for *E. coli* on average), which is consistent with the results obtained in our study (−0.03 log for MS2 and −4 log on average for *E. coli*) (Van der Laan et al., 2014). Indeed, all tested bacteriophages successfully passed through the porous candles, mainly due to their size (27 nm for MS2 and ΦX174 and 75 nm for Φ6), which is considerably smaller than 2‐µm pores. In contrast, *E. coli* K12 was significantly retained during filtration, especially due to its length (2 µm), thus proving the effective exclusion of particles larger than 2 µm. Until now, the largest viruses reported, also known as giant viruses, belong to the *Mimivirus* genus and are known to infect protists. They are non‐tail‐shaped particles with a capsid of approximately 500 nm and a genome size of 1300 kb (Drulis‐Kawa et al., 2014; Serwer et al., 2007), which are still smaller than the pores of the porous candles. Theoretically, and based on the initial results, viruses should not be affected by size exclusion when passing through porous candles. However, other phenomena may be responsible for virus loss during the filtration process through candles. Viruses have electrical charges on their surface, inducing interactions with other molecules, such as minerals. These electrical charges depend strongly on the pH of the surrounding solutions, which is linked to the pI of viruses. The first in vitro experiment was performed at a neutral pH (pH = 7.0) so that all the bacteriophages studied had a negative electrical charge on their surface. To study whether pH could cause adsorption phenomena between the bacteriophage and the candle coating, a second experiment consisted of reducing the pH of bacteriophage solutions to 4.4 so that the bacteriophages had different surface charges. At this pH value, MS2 remained negatively charged while ΦX174 was positively charged. As expected, the concentration of MS2 did not significantly decrease after candle filtration, regardless of the change in pH. In contrast, the changes in surface charges of ΦX174 triggered a slight reduction (−1.57 log), however, this was not significantly different from the feed solution.

Two main mechanisms can be considered in the reduction of ΦX174: its inactivation or adsorption. An acidic pH (≈2) tends to inactivate bacteriophages due to the denaturation of proteins or nucleic acids (Feng et al., 2003; Jurczak‐Kurek et al., 2016; Nishide et al., 2011). Furthermore, an acidic pH can also be responsible for the aggregation process. Indeed, bacteriophages tend to aggregate with each other when the pH goes below the pI (Jończyk et al., 2011; Langlet et al., 2007). This process increases the apparent size of the particles to a few micrometers, inducing potential obstacles during their passage through the filter pores. This phenomenon can be observed in a plaque assay since the aggregation of bacteriophages results in a reduction in the number of plaques (Langlet et al., 2007, 2008). However, our results (Appendix) did not reveal that a pH of 4.4 had a significant effect on the inactivation of ΦX174, supporting the adsorption hypothesis. A study on viral adsorption during filtration processes highlighted the stability of MS2 at different pH values compared to ΦX174 (Elhadidy et al., 2013). More precisely, the repulsive forces between ΦX174 and the ceramic surface were reduced as the pH decreased. The adsorption process occurs either through electrostatic forces or hydrophobic interactions (Chattopadhyay & Puls, 2000; Flynn et al., 2004; Käss, 2004; Zemb et al., 2013). The ceramic part of the candles used in this study is composed of quartz, also called silicon dioxide, formed of silicon (Si^+4^) and oxygen (O^−2^), a hydrophobic molecule. Note, MS2 is highly hydrophobic, while ΦX174 has low hydrophobicity (Farkas et al., 2015; Kreißel et al., 2012; Oudega et al., 2021). Therefore, ΦX174 will preferentially interact with the hydrophobic surface of the candle, compared to MS2. However, a slight reduction of ΦX174 was observed when the pH was changed while the hydrophobic interactions were not impacted by pH change, reinforcing potential adsorption by electrostatic interactions. The silicon (cation) is in the central position of the quartz surrounded by four oxygen atoms (anion), which makes the quartz inert and incapable of electrostatic interaction with ΦX174. However, the ceramic part is made of clay, a natural soil material composed of silica, alumina, and water, and presents a permanent negative charge (Kumari & Mohan, 2021; Mohammed et al., 2021). Since bacteriophage ΦX174 is positively charged after the change in pH, electrostatic interactions with the negative charges of the ceramic could have caused the observed reduction in ΦX174 concentration. Such interactions are well known and have particularly been documented between bacteriophages and mineral surfaces, such as saprolite (Flynn et al., 2004; McKay et al., 2000) or metal oxides (i.e., 2–4 log of loss) (Flynn et al., 2015; Kvitsand et al., 2015). Despite the slight difference observed in the reduction of ΦX174, it is important to note that this reduction was not significantly different from that of the feed solution, allowing us to assume the effectiveness of the porous candles in collecting bacteriophages, and thus, viruses smaller than 2 µm.

Soil viral communities are predominantly composed of bacteriophages, and more particularly of DNA‐bacteriophages (Emerson, 2019; Trubl et al., 2020). This study therefore focused the aim on the collection of DNA viruses. However, it should be noticed that RNA viruses are also an important part of the soil viral community, and they could be collected and detected using library preparation adapted for RNA viruses (i.e., including a retro‐transcription step). As the analysis of the Weierbach soils showed, DNA viruses were successfully collected from the soil water with the porous candles and were the second most identified population after bacteria. In particular, DNA‐bacteriophages were the most frequently found viruses with identified contigs belonging mainly to the most representative families of viral taxonomy: *Siphoviridae*, *Myoviridae*, and *Podoviridae*. The taxonomy was assigned using the nr database released in April 2022, however, it is noticeable that the viral taxonomy built by the International Committee on Taxonomy of Viruses (ICTV) is nowadays updated according to the genetic similarities between bacteriophages, including a reclassification of the families observed in the current study, such as *Siphoviridae*, *Myoviridae*, and *Podoviridae*. However, it is noteworthy that the bacterial contigs from our samples represented 90% of the total identified contigs. Among the limitations of viral metagenomics, the small size of viral genomes (i.e., up to 500 kbp) compared to bacterial genomes (i.e., up to 8 Mbp) can make viral genome reconstruction challenging during *de novo* assembly (Hatfull & Hendrix, 2011; Koduru, 2019; Rose et al., 2016). Indeed, although viruses are more abundant in environments than their hosts, the viral genomes are less represented than those of their hosts within the bulk metagenomes, which can lead to an over‐representation of bacterial contigs after shotgun metagenomics compared to that of the viruses. To mitigate this, enrichment and amplification approaches are required before extraction to increase the DNA concentration (Gaafar & Ziebell, 2020; Hall et al., 2014; Lien et al., 2007). Additionally, despite the growing number of viruses being discovered, viral genomes in the general databases (e.g., RefSeq, nr, Genbank) remain underrepresented compared to bacterial genomes (Rose et al., 2016). These databases are also dependent on research topics as they are built based on repository submission, and thus, require extensive alignment methods (Phadke et al., 2021). In this study, bioinformatics tools not specifically targeting viruses were used to identify all microbial populations (i.e., virus, bacteria, archaea…), as the detection of other microorganisms than viruses was also of interest in the study of porous candle efficiency. However, when studying viral diversity, this bioinformatics pipeline may under‐detect viruses and virus‐specific bioinformatics tools would be preferable for further analysis (Roux et al., 2015). Nevertheless, our bioinformatics pipeline did successfully detect viral populations from the collected soil water and allowed us to prove that viruses passed through the porous candles.

Most publications report on the use of ceramic filtration in microbiology, which aims to remove microbes, including viruses, during water treatment processes while their passage through the ceramic has been poorly studied (Van der Laan et al., 2014; Michen et al., 2012; Nigay et al., 2020). When using porous ceramic filters to clean water of biological pathogens, such as bacteria, parasites, or even fungi and suspended particles (e.g., turbidity, soil aggregates), viral removal has always been a challenge (Hammel et al., 2014; Lelago Bulta et al., 2019; Zereffa & Bekalo, 2017). The generally small size of viruses prevents them from being retained outside of the filter (Michen et al., 2012). This ability of viruses to pass through the pores may be beneficial for collecting them from soil water. Indeed, this method has already been used to collect trace metals, pesticides, or nitrogen in the soil (SDEC, 2002), and it may be applicable for viruses. Soil is a complex compartment with many organisms that can be detected by molecular techniques. When collecting soil water only particles smaller than 2 µm can pass through the porous candles. This can be an interesting advantage when collecting soil water for viral analyses, especially to reduce the sampling quantity of larger bacteria. Additionally, porous candles are easy to install in the soil and can be reused in new sites after rinsing, without significantly disturbing the soil structure (Curley et al., 2011). This soil water sampler works in all types of soil (i.e., clay, sandy, silty) and allows analyses at any depth at any time (Grobler et al., 2003). However, it is noteworthy that the efficiency of the device will be decreased in drier soils, and the collected volume will depend on the soil water content. Based on the results obtained in the laboratory experiments, the variability between porous candles in the recovery of bacteriophage was found to be low. However, the laboratory experiments do not take into account the complexity of the soil. First, viruses can attach to soil particulates through either electrostatic forces or hydrophobic bindings, which may interfere with the collection of viruses from soil water (Bitton, 1975; Davis et al., 2006). The attachment process in the soil is highly complex since it depends on many factors, such as the soil pH and texture, or the organic matter content, and thus, is very difficult to investigate. Then, variability in nutrient concentrations after collection with ceramic samplers has been observed (Hansen & Harris, 1975). In addition, the presence of macropores and micropores in soils results in preferential pathways for water, which are impossible to predict during collection (Curley et al., 2011). This can lead to difficulties in anticipating the volume of soil water collected, which is highly dependent on soil properties and climate conditions (Holder et al., 1991). After the collection of soil viruses, metagenomics analysis has identified viruses that were able to pass through the porous candles, but it does not provide information on their infectivity or integrity. Indeed, these viruses may be either intact or in the state of free DNA or RNA. Culture‐based tests would be needed to verify this, however, this application is difficult to implement for soil viral populations, since first, most viruses are still poorly known or not known at all, and second, most host strains are not available for detection and replication in the laboratory.

The porous candles showed very promising results in the collection of viruses from soil water since no significant loss after passage through the ceramic was observed under laboratory conditions, and natural viruses including bacteriophages were successfully detected after in situ samplings. Therefore, this new virological sampling device could open new perspectives in the study of terrestrial viral diversity and its ecological dynamics.

## 5 CONCLUSIONS

Porous ceramic candles have proven to be useful for collecting bacteriophages from liquid media in a laboratory experiment and for collecting viruses from soil water in the field. Indeed, their small size prevented the size‐exclusion of the viruses through the candles. As expected, the acidification of the solution did not affect the passage of MS2. More surprisingly, ΦX174 was also not significantly impacted, despite the change in its surface charges. However, a slight reduction in the concentration of ΦX174 was observed upon acidification of the ΦX174 solution, mostly due to adsorption by electrostatic forces between bacteriophages on the ceramic part of the candle. Ultimately, the successful collection of the viruses during the field experiment allowed us to conclude the ability and the interest of the porous candles to collect such small microorganisms from soil water.

## AUTHOR CONTRIBUTIONS

**Perrine Florent**: Conceptualization (lead); Data curation (lead); Formal analysis (lead); Investigation (lead); Methodology (lead); Writing–original draft (lead); Writing–review & editing (equal). **Henry‐Michel Cauchie**: Formal analysis (supporting); Funding acquisition (supporting); Investigation (supporting); Methodology (supporting); Supervision (supporting); Writing–original draft (supporting); Writing–review & editing (equal). **Malte Herold**: Investigation (supporting); Methodology (supporting); Software (lead). **Leslie Ogorzaly**: Conceptualization (supporting); Formal analysis (supporting); Funding acquisition (supporting); Investigation (supporting); Methodology (supporting); Supervision (lead); Validation (supporting); Writing–original draft (supporting); Writing–review & editing (equal).

## CONFLICT OF INTEREST

None declared.

## ETHICS STATEMENT

None required.

## Figures and Tables

**Figure A1:**
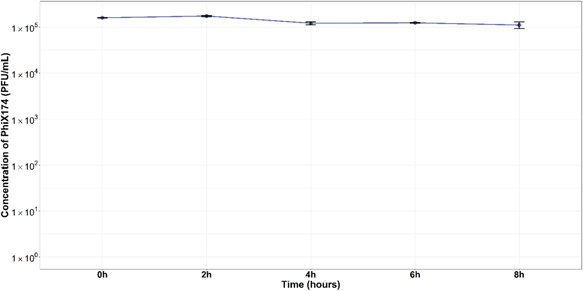
Effect of acidic pH (4.4) on ΦX174 concentration over 8 h experiment. A nonparametric test (Kruskal–Wallis One Way ANOVA on rank) for small comparisons (*n* = 2) was performed on the ΦX174 concentrations.

## Data Availability

The raw sequence data generated and analyzed in the current study are available in the National Centre for Biotechnology Information Databases (Sequence Read Archive) repository, under accession number PRJNA823538: http://www.ncbi.nlm.nih.gov/bioproject/823538.

## APPENDIX A

Effect of acidic pH on ΦX174 inactivation

Material and methods

Acidic pH (here, 4.4) could induce an indirect reduction in the concentration of ΦX174 through the adsorption process, however, pH is also known to directly affect the inactivation of ΦX174, leading to concentration reductions (Feng et al., 2003; Nishide et al., 2011; Jurczak‐Kurek et al., 2016). Therefore, to test whether pH 4.4 could cause a reduction in ΦX174 concentration, a solution of ΦX174 was prepared with peptone saline water and acidified to pH 4.4 using hydrochloric acid (HCl, pH = 3.1) to reach a concentration of 1.60 × 105 PFU mL^−1^. The concentrations of ΦX174 were determined every 2 h for a total duration of 8 h, thus covering the time of the laboratory experiment for ΦX174, using the double agar layer assay (according to the procedures of ISO 10705‐2:2001). The effect of pH on ΦX174 inactivation was tested using a nonparametric test (Kruskal–Wallis One Way ANOVA on rank) for small comparisons (*n* = 2 replicates).

**Results**

The analysis performed on the effect of pH 4.4 on ΦX174 inactivation revealed a constant concentration of ΦX174 for 8 h (Figure A1). The initial concentration of ΦX174 of 1.60 × 105 PFU mL^−1^ was reduced to 1.22 × 105 PFU ml^−1^ after 4 h and to about 1.12 × 105 PFU mL^−1^ at the end of the experiment (after 8 h). No significant difference in ΦX174 concentration was found all along the experiment (*p* = 0.1204, *α* = 0.05).
